# Rapid, reliable mobile assessment of affect-related motor processing

**DOI:** 10.3758/s13428-022-02015-y

**Published:** 2022-12-16

**Authors:** Jonathon R. Howlett, Florence Larkin, James Touthang, Rayus T. Kuplicki, Kelvin O. Lim, Martin P. Paulus

**Affiliations:** 1 VA San Diego Healthcare System, 3350 La Jolla Village Dr, San Diego, CA 92161 USA; 2 Department of Psychiatry, University of California San Diego, La Jolla, CA USA; 3 Minneapolis VA Health Care System, Minneapolis, MN USA; 4 Laureate Institute for Brain Research, Tulsa, OK USA; 5 Department of Psychiatry and Behavioral Sciences, University of Minnesota, Minneapolis, MN USA

**Keywords:** Computational psychiatry, Mobile assessment, Motor control, Anxiety, Affect, Inhibitory control

## Abstract

Mobile technologies can be used for behavioral assessments to associate changes in behavior with environmental context and its influence on mental health and disease. Research on real-time motor control with a joystick, analyzed using a computational proportion-derivative (PD) modeling approach, has shown that model parameters can be estimated with high reliability and are related both to self-reported fear and to brain structures important for affective regulation, such as the anterior cingulate cortex. Here we introduce a mobile version of this paradigm, the rapid assessment of motor processing (RAMP) paradigm, and show that it provides robust, reliable, and accessible behavioral measurements relevant to mental health. A smartphone version of a previous joystick sensorimotor task was developed in which participants control a virtual car to a stop sign and stop. A sample of 89 adults performed the task, with 66 completing a second retest session. A PD modeling approach was applied to compute *K*_*p*_ (drive) and *K*_*d*_ (damping) parameters. Both *K*_*p*_ and *K*_*d*_ exhibited high test-retest reliabilities (ICC .81 and .78, respectively). Replicating a previous finding from a different sample with the joystick version of the task, both *K*_*p*_ and *K*_*d*_ were negatively associated with self-reported fear. The RAMP paradigm, a mobile sensorimotor assessment, can be used to assess drive and damping during motor control, which is robustly associated with subjective affect. This paradigm could be useful for examining dynamic contextual modulation of affect-related processing, which could improve assessment of the effects of interventions for psychiatric disorders in a real-world context.

**Supplementary Information:**

The online version contains supplementary material available at 10.3758/s13428-022-02015-y.

## Introduction

Despite major advances in technology, psychiatric research continues to confront a lack of accurate and reliable markers of core underlying process dysfunctions (Pine & Leibenluft, 2015). In particular, three key challenges face attempts to improve individualized, mechanistic assessments in psychiatry: (1) difficulty bridging units of analysis in light of the complexity of relationships between neural functions, behavior, and subjective experience (Insel et al., 2010); (2) poor statistical reliability of many common individual behavioral and neural measures of core constructs relevant to psychiatric illness (Enkavi et al., 2019); (3) reliance on retrospective self-report, which may conceal the important dynamic contextual modulation of disease processes (Shiffman et al., 2008). Overcoming these challenges by developing reliable, repeatable assessments which correlate with underlying disease processes could be an important step toward more effectively assessing treatments for psychiatric disorders.

Incorporation of emerging mobile digital technologies such as smartphones and other devices into psychiatric assessments represents a major opportunity to gather large amounts of data in real-world settings, potentially enabling robust, cost-effective, and highly accessible assessments of outcome-relevant behavioral variables. While introducing new challenges in terms of usability engineering, data security, informed consent, and cultural and language issues (Holmlund et al., 2019), smartphone applications to monitor psychiatric illnesses have been successfully investigated for mood disorders, anxiety disorders, substance use disorders, and others (Swendsen & Salamon, 2012). The array of high-quality sensors embedded in modern mobile devices offer the opportunity for collection of large amounts of data and could enable the design of sophisticated experimental paradigms to measure mechanistic behavioral dysfunction in a real-world setting in a robust manner (Torous et al., 2015). Additionally, mobile devices offer a convenient platform for collection of self-report data in real-time, an approach known as ecological momentary assessment (EMA) (Shiffman et al., 2008). Mobile technology therefore has the potential to address multiple challenges in psychiatric assessment by collecting rich information across units of analysis in a real-world context.

To leverage the potential for mobile assessments to link self-report and behavioral data to guide treatment decisions, reliable behavioral markers of processes relevant to psychiatric disorders are necessary. Recently, large scale analyses have revealed that an array of common behavioral paradigms measuring self-regulation or control exhibit poor reliability (Enkavi et al., 2019), which would hamper their usefulness as clinical assessments. A possible solution is to move beyond discrete choice and reaction time data and instead analyze continuous, real-time data to more reliably measure individual differences in control processes. We previously developed a simple simulated driving task in which a subject drives a virtual car to a stop sign and then stops (Howlett et al., 2020). This task requires dynamic motor control to prevent the car from driving past the stop sign, but yields dense real-time continuous data (with 60 data points collected per second) unlike many tasks probing control processes (e.g., those requiring a single button press per trial). We applied a proportion-derivative (PD) control framework which includes a proactive drive component (a *K*_*p*_ parameter) and a reactive damping component (a *K*_*d*_ parameter). PD control is a general framework which can be applied to many different control scenarios (Howlett et al., 2020; Johnson & Moradi, 2005). The drive component causes acceleration in proportion to the current distance from a goal state. The damping component, which is analogous to friction in a physical system, enables slowing in proportion to the current velocity and prevents overshoot past the goal state (for further details, see Howlett et al., 2020). In a transdiagnostic sample of individuals with mood and anxiety complaints as well as healthy controls, we found that low *K*_*p*_ and low *K*_*d*_ were associated with self-reported fear and with low volume of the dorsal anterior cingulate cortex (dACC), as well as female gender and older age. Model parameters were therefore related to both self-reported affect and to affect-related circuitry. PD model parameters were estimated with extremely high split-half reliability (*r* = .98 for *K*_p_ and *r* = .95 for *K*_d_) and high reliability even for small numbers of trials. While the original version of the task used a gaming joystick and laptop computer, the touch screen capabilities of mobile electronic devices present the opportunity to adapt it to a mobile context, enabling the probing of control processes linked to affect outside of a laboratory setting. Once validated, a mobile version of the task could allow for behavioral data collection in parallel with self-report and potentially other data sources to improve measurement of dynamic changes in control performance in a real-world context.

This manuscript describes the development and validation of a mobile version of the previous joystick driving task (Howlett et al., 2020). We developed the rapid assessment of motor processing (RAMP) paradigm, a version of the task in which subjects control car velocity using a thumb on the device touch screen. We tested the task in a sample of individuals recruited from Amazon’s Mechanical Turk in a test-retest design. We hypothesized that we would replicate our findings from the joystick version of the task, demonstrating high test-retest reliability as well as relationships between model parameters and self-reported fear, along with gender and age.

## Method

### Participants

Adults age 18 and over were recruited via Amazon’s Mechanical Turk to participate in two remote experimental sessions. Participants were compensated $2 for the first session and $4 for the second session. The second session was available as soon as the first session was complete and expired after 7 days. See Supplemental Table 1 for the distribution of days between experimental sessions. Eighty-nine participants (age: 35.78 ± 9.54, range 23–68; gender: 57 male and 32 female; see Table 1) completed session 1, and of these, 66 (age: 36.05 ± 10.30; gender: 44 male and 22 female) completed session 2. While participants were instructed to use mobile devices for the task, nine participants in session 1 and five participants in session 2 used non-mobile devices (potentially using a mouse or trackpad instead of a touch screen input). All study procedures were approved by the Western Institutional Review Board, and all participants provided written informed consent prior to participation.

**Table 1:** Participant characteristics

	Mean	SD
Demographics		
Gender (% male)	64.0	
Age	35.8	9.5
Education (%)		
Less than high school	3.4	
High school or GED	7.9	
Some college	10.1	
Bachelor’s degree or higher	78.7	
Region (%)		
North America	71.9	
South America	11.2	
Asia	11.2	
Europe	5.6	
Race/ethnicity (%)		
White	52.8	
Asian	15.7	
Hispanic	14.6	
Black	12.4	
American Indian	3.4	
No answer	1.1	

### Task development

We developed the RAMP paradigm, a mobile version of a previously published joystick driving task (Howlett et al., 2020), to assess individual differences in indices of PD control, representing real-time proactive and reactive inhibitory control. The mobile version of the driving task was developed using Flutter (https://flutter.dev), which is an open-source framework for developing applications created by Google. Flutter can be used to create applications across multiple platforms including Android, iOS, and browser apps. Flutter applications are written in the Dart programming language. We chose to create a JavaScript browser-based task to avoid barriers in creating a mobile application available across multiple platforms.

Several challenges were addressed in the process of developing a mobile version of the joystick task. First, multiple input options were considered, including the use of the mobile device’s accelerometer, before touch screen input was chosen as the control was intuitive and most closely matched the joystick control. Despite the relative similarity, differences between the two input methods included the fact that a joystick has a return-to-center feature. Different screen sizes across devices also needed to be considered (see Supplemental Methods for more details). The development process involved multiple task iterations which were tested first locally and later on Amazon’s Mechanical Turk. The task took a year to develop.

### Experimental task

**Fig. 1:**
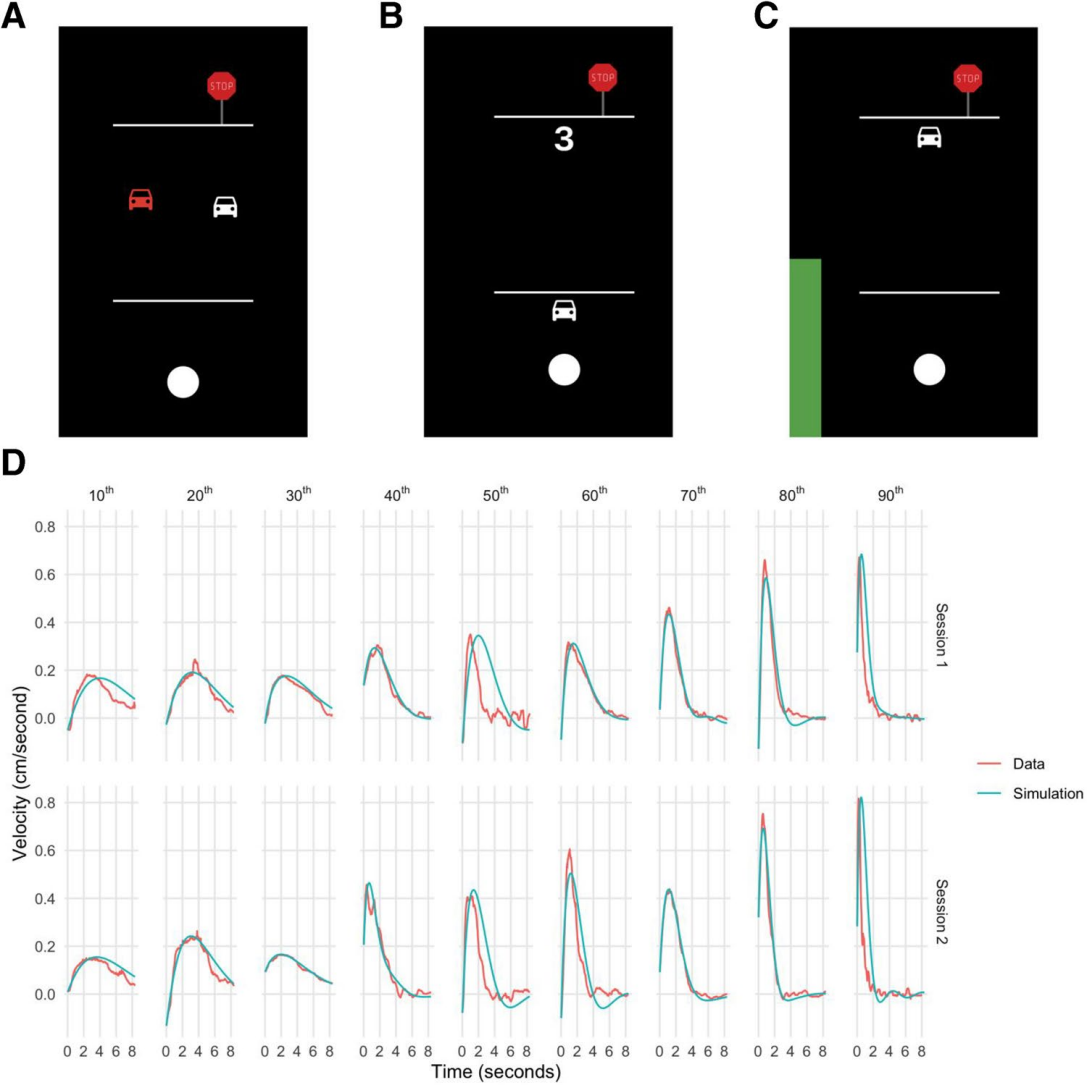
Experimental task and model. (**A**) Practice session. Two cars appeared on the screen, of which one was controlled by the participant and the other moved automatically forward and backward. Participants were instructed to follow parallel to the demonstration car as closely as possible to practice controlling the car. (**B**) Task countdown. (**C**) Experimental trial. Participants were instructed to drive as quickly as possible to the stop sign and then remain stopped. (**D**) Data and model fit. Ten participants were selected based on percentile *K*_*p*_ value (10^th^, 20^th^, 30^th^, 40^th^, 50^th^, 60^th^, 70^th^, 80^th^, and 90^th^) computed from the first experimental session. Data traces were generated by averaging the velocity trajectories across all trials for experimental sessions 1 and 2. Simulation traces were generated by simulating a velocity trajectory for each trial using the PD model based on computed *K*_*p*_ and *K*_*d*_ parameters, then averaging the traces across trials

Participants were given a link to the task on Mechanical Turk and instructed to open the link on their mobile device to ensure participants were not using a mouse or a laptop trackpad to complete the task. For the experimental task, participants controlled a simulated car in one dimension by holding a thumb on an area of the device touch screen (indicated by a white circle) and sliding the thumb forward to accelerate and backward to decelerate the car. The task is not meant to represent a realistic driving experience (e.g., with a separate accelerator and brake) but instead to probe real-time motor control processing. Participants were first provided with instructions and completed a brief practice session (see Fig. 1A) to become familiar with the control mechanism. In this session, two cars appeared on the screen, of which one was controlled by the participant and the other moved automatically forward and backward. Participants were instructed to follow parallel to the demonstration car as closely as possible to practice controlling the car. The practice trial lasted 30 seconds and was restarted if the participant was unable to maintain the controlled car within a certain distance of the target car. Once participants passed the practice trial, they moved on to the actual task.

The task was formatted as two blocks of 10 trials, with each trial lasting about 10 seconds. The position of the car and the instantaneous velocity were recorded automatically at every frame. Each trial was preceded by a countdown (see Fig. 1B), after which participants were instructed to control the car to a stop sign as quickly as possible (see Fig. 1C) and then stay stopped at the stop sign for the remainder of the trial. Participants were instructed to hold the thumb to the touch screen throughout the duration of trial, and trials were restarted if the thumb was removed from the touch screen, if the car was driven off the screen (i.e., well beyond the stop sign), or if the car was not within a certain distance of the stop sign at the end of the trial. If the subject completed the trial successfully, they moved on to the subsequent trial. In both the original version of the task using the joystick, and this version, the time for the car to reach the stop sign at maximum speed was 0.75 seconds. Excluding the practice trial, the duration of the task was typically 5–6 minutes.

### Self-report questionnaire

Prior to the experimental task, participants completed a demographic questionnaire and the Positive and Negative Affect Schedule-Expanded Form (PANAS X) (Watson & Clark, 1999). This questionnaire was administered in order to test the hypothesis that model parameters indexing inhibitory control (see PD Control Model subsection) would be negatively related to self-reported fear, as was the case for the previous version of the task (Howlett et al., 2020).

### PD control model

For each trial, we fit a PD control model to estimate *K*_*p*_ and *K*_*d*_ parameters. Model fitting was performed using linear regressions in R (R Core Team, 2013). In the PD control framework, acceleration toward a goal state (i.e., the stop sign) is controlled based on both distance to the goal and the derivative of the distance (i.e., velocity). *K*_*p*_ is a weighting factor which drives acceleration toward the goal based on the current distance, while *K*_*d*_ is a weighting factor which causes deceleration in proportion to the current velocity, preventing overshoot past the goal state. *K*_*d*_ can be considered as a damping term as it is analogous to friction in a physical system. From a stopped position, the drive term will initially outweigh the damping term causing acceleration, but as velocity increases and distance decreases, the damping term will outweigh the drive term, causing deceleration and preventing overshoot. Increased *K*_*d*_ can be considered as a form of reactive inhibitory control, since it inhibits movement in response to incoming information. Decreased *K*_*p*_ can be considered a form of proactive inhibitory control, since reducing initial drive will also reduce the tendency to overshoot the goal state.

At each time point within a trial, acceleration was modeled as a dependent variable with error (goal position minus current car position) and derivative of the error as predictors, and with an intercept of zero. The coefficients of the error and derivative terms were used as estimates of *K*_*p*_ and *K*_*d*_, respectively. Model fits were inspected visually by comparing the velocity trace predicted by the fitted *K*_*p*_ and *K*_*d*_ values for each trial with the observed velocity trace. Additionally, in order to assess model fits, we simulated each trial from starting conditions using the PD model with estimated *K*_*p*_ and *K*_*d*_ parameters, and then computed the *R*-squared statistic between predicted velocity and observed velocity at each time point throughout the trial, to determine the variance in velocity explained by the model prediction. Log *K*_*p*_ and *K*_*d*_ values were used in analyses as visual inspection revealed these parameters were right skewed across participants (see Supplemental Figure 1).

### Reliability of parameter estimation

To assess the reliability of parameter estimation within each experimental session, we separately computed mean log *K*_*p*_ and log *K*_*d*_ values for odd and even trials for each session for each subject. We computed Pearson correlations between odd-trial and even-trial estimates for log *K*_*p*_ and log *K*_*d*_ separately for session 1 and session 2. We additionally performed the same calculations except splitting trials by first and second half of the experimental session rather than odd and even trials. We also performed Fisher’s *z* transformation to determine whether correlations differed between session 1 and session 2.

To assess test-retest reliability of parameter estimation across experimental sessions, we calculated mean log *K*_*p*_ and log *K*_*d*_ values for each experimental session in participants who completed both sessions. We then computed intraclass correlation coefficients (ICC3 based on a single fixed rater model) for log *K*_*p*_ and log *K*_*d*_ using R. We also computed ICC values excluding participants who completed both experimental sessions on the same day as well as excluding participants who did not use a mobile device.

### Relationship between model parameters and individual characteristics

To examine the relationship between model parameters and demographic variables, we constructed linear regression models using data from session 1 with log *K*_*p*_ or log *K*_*d*_ as the dependent variable and with age, gender, and education as predictors. Variables were scaled to enable estimation of standardized regression coefficients. Given the small number of participants in the less than high school, high school or GED, and some college categories, we treated education as a dichotomous variable (some college or less and bachelor’s degree or higher) in all models.

**Fig. 2:**
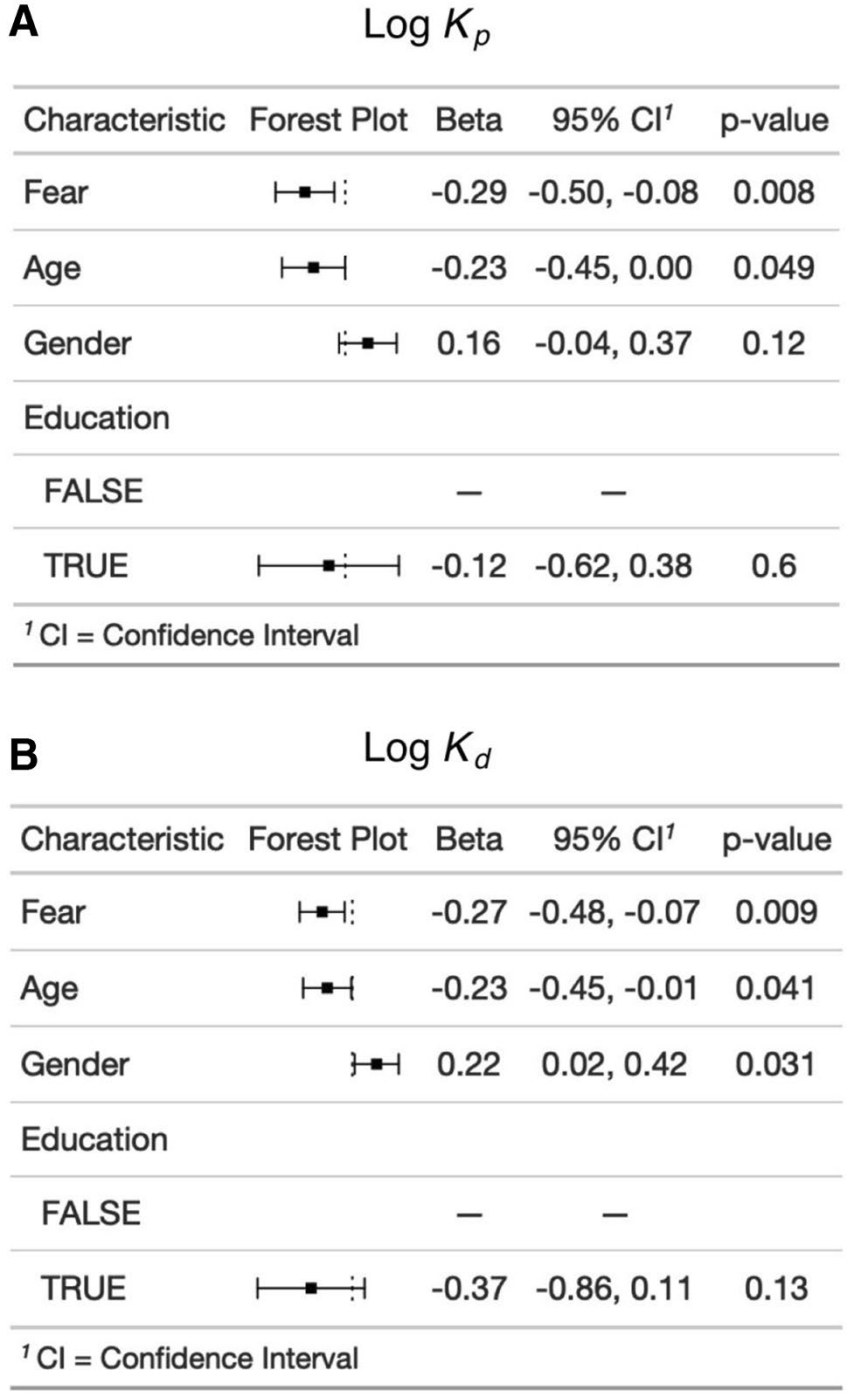
Relationships between model parameters and individual characteristics. (**A**) A linear regression model was performed with log *K*_*p*_ as dependent variable and PANAS X Fear score, age, male gender, and education as predictors. Variables were scaled to generate standardized beta values. (**B**) Identical model except with log *K*_*d*_ as dependent variable

To determine whether model parameters were related to self-reported fear as hypothesized based on findings from the non-mobile driving task (Howlett et al., 2020), we additionally constructed linear regression models with log *K*_*p*_ or log *K*_*d*_ as the dependent variable and with PANAS X Fear score along with age, gender, and education as predictors (see Fig. 2). Variables were scaled to enable estimation of standardized regression coefficients.

We also examined the relationship between PANAS X Fear score and non-model-based indices of task performance. We constructed linear regression models with final car position or log of the number of trials terminated due to errors as dependent variables and with PANAS X Fear score along with age, gender, and education as predictors.

## Results

### Reliability of parameter estimation

The PD model provided a good fit to data based on visual inspection of velocity traces (see Fig. 1D). Across all trials, the mean *R*-squared between predicted and observed velocity was .70 (sd .23), indicating the model prediction based only on the two PD model parameters explained about 70% of the observed variance in velocity measurements across all time points within a trial. In session 1, the correlation between log *K*_*p*_ estimated from odd trials and log *K*_*p*_ estimated from even trials was .95 (*p* < .001) while the correlation between log *K*_*p*_ estimated from the first half of trials and log *K*_*p*_ estimated from the second half of trials was .87 (*p* < .001). The correlation between log *K*_*d*_ estimated from odd trials and log *K*_*d*_ estimated from even trials was .89 (*p* < .001), while the correlation between log *K*_*d*_ estimated from the first half of trials and log *K*_*d*_ estimated from the second half of trials was .81 (*p* < .001). In session 2, the correlation between log *K*_*p*_ estimated from odd trials and log *K*_*p*_ estimated from even trials was .92 (*p* < .001), while the correlation between log *K*_*p*_ estimated from the first half of trials and log *K*_*p*_ estimated from the second half of trials was .90 (*p* < .001). The correlation between log *K*_*d*_ estimated from odd trials and log *K*_*d*_ estimated from even trials was .82 (*p* < .001), while the correlation between log *K*_*d*_ estimated from the first half of trials and log *K*_*d*_ estimated from the second half of trials was .77 (*p* < .001). Correlations did not differ between session 1 and session 2 for log *K*_*p*_ based on odd/even split (*z* = 1.35, *p* = .18) or first/second half (*z* = −0.64, *p* = .52) or for log *K*_*d*_ based on odd/even split (*z* = 1.57, *p* = .12) or first/second half (*z* = 0.56, *p* = .58).

The ICC for log *K*_*p*_ between session 1 and session 2 was .84 (*p* < .001). The ICC for log *K*_*d*_ between session 1 and session 2 was .78 (*p* < .001). Excluding the seven participants who completed both sessions on the same day, the ICC for log *K*_*p*_ between session 1 and session 2 was .87 (*p* < .001) and the ICC for log *K*_*d*_ between session 1 and session 2 was .80 (*p* < .001). Excluding the six participants who did not use mobile devices for both sessions, the ICC for log *K*_*p*_ between session 1 and session 2 was .84 (*p* < .001) and the ICC for log *K*_*d*_ between session 1 and session 2 was .76 (*p* < .001).

### Relationship between model parameters and individual characteristics

In linear regression models using data from session 1 (89 participants), log *K*_*p*_ was not significantly associated with male gender (*β* = .19, *p* = .08), age (*β* = −.14, *p* = .20), or education (bachelor’s degree or higher: *β* = −.09, *p* = .74). Log *K*_*d*_ was positively associated with male gender (*β* = .25, *p* = .02) but was not significantly associated with age (*β* = −.12, *p* = .26) or education (bachelor’s degree or higher: *β* = −.34, *p* = .18). In session 2 (66 participants), log *K*_*p*_ was not significantly associated with male gender (*β* = .15, *p* = .23), age (*β* = −.09, *p* = .46), or education (bachelor’s degree or higher: *β* = .99, *p* = .55). Log *K*_*d*_ was not significantly associated with male gender (*β* = .10, *p* = .45), age (*β* = −.06, *p* = .66), or education (bachelor’s degree or higher: *β* = .02, *p* = .96).

During session 1 (89 participants), log *K*_*p*_ was negatively associated with PANAS X Fear score (*β* = −.29, *p* = .006) controlling for age, gender, and education. Log *K*_*d*_ was also negatively associated with PANAS X Fear score (*β* = −.29, *p* = .006) controlling for age, gender, and education. Excluding the nine participants who did not use a mobile device, log *K*_*p*_ was negatively associated with PANAS X Fear score (*β* = −.33, *p* = .002) controlling for age, gender, and education. Log *K*_*d*_ was also negatively associated with PANAS X Fear score (*β* = −.31, *p* = .003) controlling for age, gender, and education.

During session 2 (66 participants), the relationship between log *K*_*p*_ and PANAS X Fear score did not reach significance (*β* = −.23, *p* = .08) controlling for age, gender, and education. Similarly, the relationship between log *K*_*d*_ and PANAS X Fear score did not meet significance (*β* = −.24, *p* = .07) controlling for age, gender, and education. However, excluding the five participants who did not use a mobile device, log *K*_*p*_ was negatively associated with PANAS X Fear score (*β* = −.28, *p* = .04) controlling for age, gender, and education. Log *K*_*d*_ was also negatively associated with PANAS X Fear score (*β* = −.29, *p* = .04) controlling for age, gender, and education.

PANAS X Fear score was not significantly related to the final car position (*β* = −.14, *p* = .18) or to the log of the number of trials terminated due to errors (*β* = .17, *p* = .12).

## Discussion

We developed the RAMP paradigm, a mobile version of our previous joystick driving task (Howlett et al., 2020) and recruited a validation sample of individuals through Amazon Mechanical Turk for test-retest sessions. Using a PD control model, we estimated *K*_*p*_ and *K*_*d*_ parameters, with higher *K*_*d*_ (a damping term) representing a form of reactive inhibitory control and lower *K*_*p*_ (a drive term) representing a form of proactive inhibitory control. Our main findings were that model parameters estimated the mobile task version exhibited high split-half (*r* = .82–.95) and test-retest (ICC = .78–.84) reliabilities and that *K*_*p*_ and *K*_*d*_ model parameters were negatively associated with self-reported fear, replicating our previous results from the joystick version. While the relationships between model parameters and fear did not reach significance in the second experimental session (possibly due to reduced power given a substantially lower number of participants that completed the second session), the relationships were significant when individuals who did not perform the task on a mobile device were excluded. In the present sample, *K*_*d*_ was positively associated with male gender, as in our previous findings. Unlike in our previous results, *K*_*p*_ was not significantly associated with gender, and neither parameter was significantly associated with age, a difference which may be attributable to a smaller sample in the present study, differences in the tasks, or to sample differences given that the previous study included individuals who were seeking treatment for mental health symptoms. Taken together, our findings suggest that the mobile driving task can serve as a reliable marker of affect-related differences in control processes in a non-laboratory setting. The ability to collect a large number of data points in a short time, coupled with the availability of mobile devices, could enable assessments that are robust, cost-effective, and highly accessible.

The last three decades have witnessed increasing interest in the use of the Internet and related technologies in delivering clinical assessments and interventions (Boogerd et al., 2015). Mobile technologies have greatly facilitated the use of self-report EMA methods to improve validity in measurement of behaviors related to substance abuse, chronic pain, physical activity, and eating behaviors (Burke et al., 2017). In parallel, recent technological advances have enabled the development of new, inexpensive “biobehavioral” measures of objective behaviors related to neurobiological functions which can be self-administered in ambulatory settings (Cohen et al., 2021). Such measures may use portable electroencephalography, eye-tracking, and facial and speech analysis in addition to accelerometers and touch screen functionality and can yield temporally dense, continuous data streams in ecologically valid settings (Cohen et al., 2021). The use of data gathered by digital sensors, integrated with self-report and clinical observation, promises to improve data-driven clinical decision making and clinical outcomes (Hsin et al., 2018) through a process that has been termed digital phenotyping (Torous et al., 2017). Traditional behavioral measures such as the Stroop task have also been successfully implemented on mobile devices (Holmlund et al., 2019). Within this context, our findings suggest that our mobile sensorimotor assessment yields highly reliable, objective individual measurements of a core control process linked to neurobiological circuit function and to affective state.

Recent research has highlighted the need for a focus on reliability of behavioral markers of individual differences. Traditional cognitive tasks are often designed to maximize the robustness of task effects at the group level, which may detract from the reliability of individual measurements (Hedge et al., 2018). A large-scale analysis of the reliability of an array of behavioral tasks probing self-regulation found that median ICC was 0.311 (−0.091 for the first quartile, 0.665 for the third quartile) (Enkavi et al., 2019), indicating that our task reliability of ICC .78–.84 is unusually high. The explanation may relate to the density of touch screen data collected with the mobile device, in which approximately 60 samples are collected each second. Therefore, a 10-second trial yields approximately 600 data points, while for many traditional tasks each trial yields a single binary choice or reaction time. Compounding this, many traditional tasks rely on differences in reaction times across conditions, which are known to be less reliable than the individual components (Hedge et al., 2018). Beyond reliability, the effect size of the relationship between model parameters and self-reported affect is also relatively high. Literature reviews have found that the relationships between behavioral measures and self-report scores across multiple domains are weak or nonexistent, with average correlations ranging from 0 to .2 (Dang et al., 2020). Our standardized *β* values of .29 for the relationship between self-reported fear and model parameters are therefore unusually high, especially considering that they are replications of a previous finding.

Future directions include the use of the mobile driving platform in natural experiments. As in previous EMA studies focused on self-report (Burke et al., 2017), individuals could receive prompts asking them to complete a small number of trials of the RAMP task at various times, yielding information about how control processes change within individuals over time. For example, studies can measure the effect of stressful events such as taking a test or going to an interview on PD control parameters. This type of natural “stress test” examining contextual modulation of affect-related control processes could reveal important aspects of disease processes which are obscured when only performing baseline assessments. These dynamic assessments could also be useful in treatment settings, such as psychotherapy, or in testing the effects of biological interventions in a real-world setting. The mobile driving platform could also be useful in measuring the effect of states other than affect on control processes, such as sleep deprivation and intoxication.

We found that a browser-based mobile task could be conveniently deployed to participants outside of a laboratory setting. The process of adapting the task required multiple iterations, particularly given the differences in input modality (joystick vs. touch screen). The successful development of a mobile version of a behavioral task could be replicated for other tasks. The ability to transfer behavioral tasks to mobile platforms could improve dynamic assessments of mental health disorders and interventions.

While the ability to collect data remotely using a mobile device is a strength of the study, the use of a sample recruited via Amazon Mechanical Turk could be considered as a limitation. However, previous research has shown that valid results can be obtained using this method (Gillan et al., 2016). Another limitation is that the RAMP task has not yet been tested in a patient population, so it is currently unknown whether it will exhibit the same reliability in those populations. Another limitation is that some individuals did not complete the task on a mobile device. The relationships between model parameters and fear appeared larger when these participants were excluded, although the number of non-mobile participants was too small to examine whether there was a significant interaction. In the future, the task could be amended to ensure that participants use a mobile device, rather than relying on instructing them to use mobile devices. Overall, our results suggest that the mobile driving platform can serve as a reliable measure of individual differences in control processes potentially relevant to psychiatric disorders, and can be administered conveniently via mobile devices in a real-world setting. Such mobile assessments could be a highly useful tool for assessing novel interventions for psychiatric disorders.

In conclusion, we developed a motor control task for mobile devices. In a validation sample, the task exhibited high reliability and relationship to self-reported fear, replicating a previous finding from a non-mobile version of the task. An assessment of control processes with high reliability, which links to other units of analysis (i.e., subjective affect) and can be deployed in a real-world context could be useful for evaluating new interventions and for examining dynamic contextual modulation of pathophysiology in psychiatric disorders.

### Supplementary Information

ESM 1 (DOCX 263 kb)

## Data Availability

The task can be accessed at https://github.com/florencelarkin/ramp_task and tested at https://florencelarkin.github.io/ramp_task/#/. Code for model fitting can be accessed at https://github.com/florencelarkin/kp_kd_fit. The data for the experiments reported will be made available in a data repository, and the code for the task and for model fitting are available on GitHub. The experiments were not preregistered.

## References

[CR1] Boogerd EA, Arts T, Engelen LJ, van de Belt TH (2015). "What is eHealth": Time for an update?. JMIR Research Protocols.

[CR2] Burke LE, Shiffman S, Music E, Styn MA, Kriska A, Smailagic A, Siewiorek D, Ewing LJ, Chasens E, French B, Mancino J, Mendez D, Strollo P, Rathbun SL (2017). Ecological momentary assessment in behavioral research: Addressing technological and human participant challenges. Journal of Medical Internet Research.

[CR3] Cohen AS, Cox CR, Tucker RP, Mitchell KR, Schwartz EK, Le TP, Foltz PW, Holmlund TB, Elvevåg B (2021). Validating biobehavioral Technologies for use in clinical psychiatry. Frontiers in Psychiatry.

[CR4] Dang J, King KM, Inzlicht M (2020). Why are self-report and behavioral measures weakly correlated?. Trends in Cognitive Sciences.

[CR5] Enkavi AZ, Eisenberg IW, Bissett PG, Mazza GL, MacKinnon DP, Marsch LA, Poldrack RA (2019). Large-scale analysis of test-retest reliabilities of self-regulation measures. Proceedings of the National Academy of Sciences of the United States of America.

[CR6] Gillan, C. M., Kosinski, M., Whelan, R., Phelps, E. A., & Daw, N. D. (2016). Characterizing a psychiatric symptom dimension related to deficits in goal-directed control. *Elife, 5*. 10.7554/eLife.11305 10.7554/eLife.11305 PMC4786435 26928075

[CR7] Hedge C, Powell G, Sumner P (2018). The reliability paradox: Why robust cognitive tasks do not produce reliable individual differences. Behavior Research Methods.

[CR8] Holmlund TB, Foltz PW, Cohen AS, Johansen HD, Sigurdsen R, Fugelli P, Bergsager D, Cheng J, Bernstein J, Rosenfeld E, Elvevåg B (2019). Moving psychological assessment out of the controlled laboratory setting: Practical challenges. Psychological Assessment.

[CR9] Howlett JR, Thompson WK, Paulus MP (2020). Computational evidence for underweighting of current error and overestimation of future error in anxious individuals. Biology Psychiatry Cognitive Neuroscience Neuroimaging.

[CR10] Hsin H, Fromer M, Peterson B, Walter C, Fleck M, Campbell A, Varghese P, Califf R (2018). Transforming psychiatry into data-driven medicine with digital measurement tools. NPJ Digital Medicine.

[CR11] Insel T, Cuthbert B, Garvey M, Heinssen R, Pine DS, Quinn K, Sanislow C, Wang P (2010). Research domain criteria (RDoC): Toward a new classification framework for research on mental disorders. The American Journal of Psychiatry.

[CR12] Johnson MA, Moradi MH (2005). *PID control*.

[CR13] Pine DS, Leibenluft E (2015). Biomarkers with a mechanistic focus. JAMA Psychiatry.

[CR14] R Core Team. (2013). *R: A language and environment for statistical computing*. R Foundation for Statistical Computing. http://www.R-project.org/

[CR15] Shiffman S, Stone AA, Hufford MR (2008). Ecological momentary assessment. Annual Review of Clinical Psychology.

[CR16] Swendsen J, Salamon R (2012). Mobile technologies in psychiatry: Providing new perspectives from biology to culture. World psychiatry : official journal of the World Psychiatric Association (WPA).

[CR17] Torous, J., Onnela, J. P., & Keshavan, M. (2017, Mar 7). New dimensions and new tools to realize the potential of RDoC: Digital phenotyping via smartphones and connected devices*. Translation Psychiatry, 7*(3), e1053. 10.1038/tp.2017.25 10.1038/tp.2017.25 PMC5416670 28267146

[CR18] Torous J, Staples P, Onnela J-P (2015). Realizing the potential of Mobile mental health: New methods for new data in psychiatry. Current Psychiatry Reports.

[CR19] Watson, D., & Clark, L. A. (1999). *The PANAS-X: Manual for the positive and negative affect schedule-expanded form*.

