# Supplementary material for: Rapid, reliable mobile assessment of affect-related motor processing
Source: Behav Res Methods. 2022 Dec 16;55(8):4260–8. doi: 10.3758/s13428-022-02015-y (PMC10700410; doi:10.3758/s13428-022-02015-y)
Supplement: Supplementary file 1 — (DOCX 263 kb) [file 13428_2022_2015_MOESM1_ESM.docx]

**Supplemental Methods**

**Experimental Task**

The car is controlled by a slider that has values ranging from -1.0 to 1.0. Participants start with their finger on the middle of the slider at the "0" position. Approximately every 16.667ms (using a periodic timer) the current position of the slider is sampled along with the position of the car in pixels and the time since starting the task. The previous time and position is also recorded so that change in position and change in time can be used to calculate the velocity of the car. To account for differences in screen sizes between mobile devices, the "adjusted position" is calculated by virtualizing the units and scaling the position value by percentage of screen size instead of pixel count. In the original version of the task using the joystick, it took 0.75 at max velocity to reach the stop sign. We used this value to scale the change in time in the calculation of the adjusted position. The final velocity is calculated by change in "adjusted position" divided by change in time divided by 1000. The function outputs a list of the current actual position (in pixels), the current adjusted position, and the velocity. The first two values are used to calculate the next position and the instantaneous velocity is recorded.

**PD Model**

Prior to model fitting, the portion of each trial prior to car movement was discarded (with movement threshold of 0.001 slider units). Frames with anomalously short sampling windows (less than 0.001 seconds) and with outlier acceleration values (less than or equal to -10 and greater than or equal to 10 units) were also discarded.

Supplemental Table 1: Days Between Sessions

| **Days Between Sessions** | **Number of Subjects** |
| --- | --- |
| 0 | 7 |
| 1 | 33 |
| 2 | 10 |
| 3 | 12 |
| 4 | 2 |
| 5 | 2 |

Supplemental Table 2: Number of Practice Trials, Session 1

| **Number of Practice Trials Needed** | **Number of Subjects** |
| --- | --- |
| 0 | 1 |
| 1 | 15 |
| 2 | 16 |
| 3 | 10 |
| 4 | 5 |
| 5 | 8 |
| 6 | 3 |
| 7 | 4 |
| 8 | 7 |
| 9 | 2 |
| 10 | 2 |
| 11 or more | 14 |

Supplemental Table 3: Number of Practice Trials, Session 2

| **Number of Practice Trials Needed** | **Number of Subjects** |
| --- | --- |
| 0 | 0 |
| 1 | 28 |
| 2 | 23 |
| 3 | 3 |
| 4 | 3 |
| 5 | 2 |
| 6 | 1 |
| 7 | 2 |
| 8 | 0 |
| 9 | 0 |
| 10 | 0 |
| 11 or more | 4 |

Supplemental Figure 1


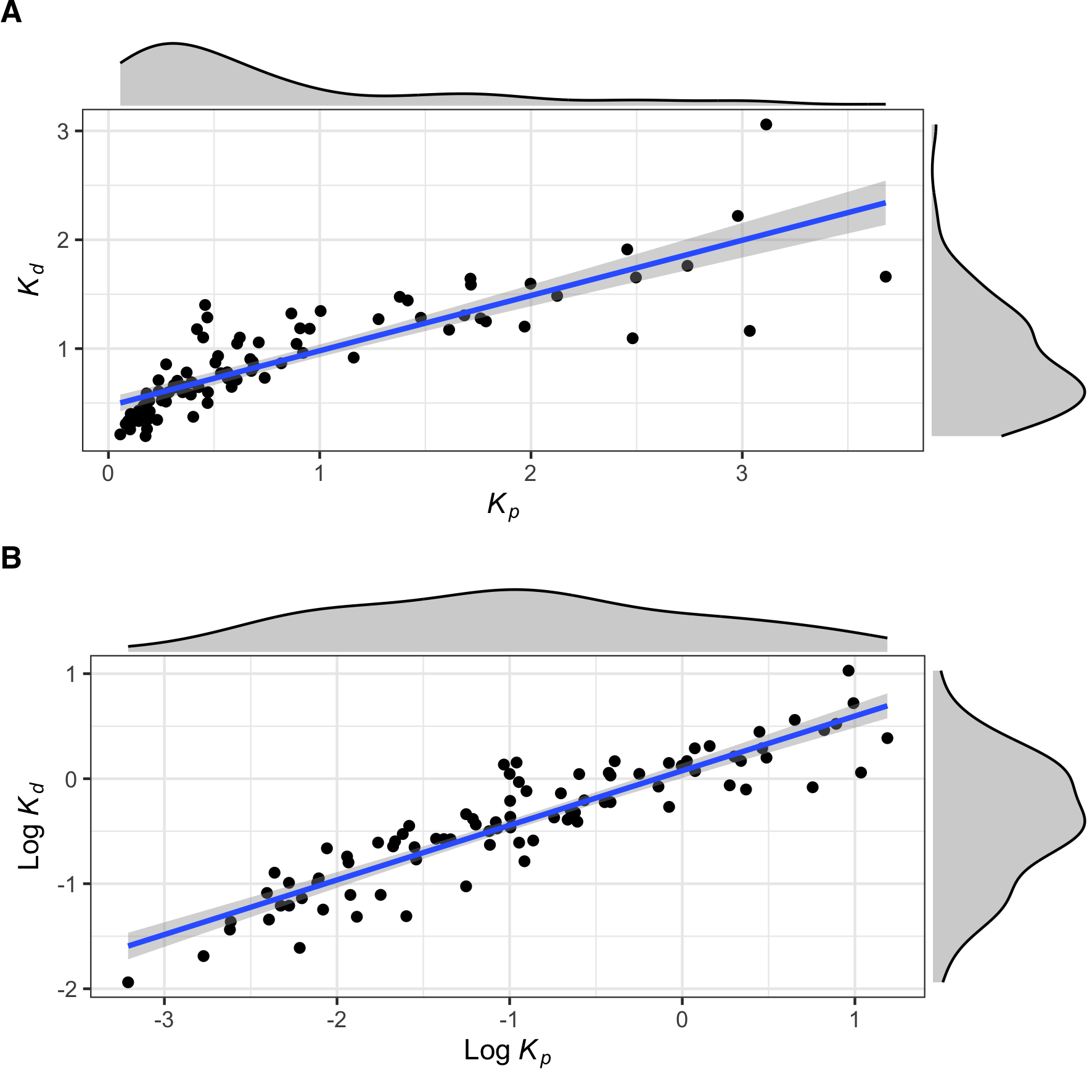


Supplemental Figure 1: Parameter distributions.

(A) Scatterplot of computed *K_p_* and *K_d_* parameters with marginal density plots for each subject in session 1. Best-fitting linear regression line is shown. Parameter distributions were right-skewed.

(B) Scatterplot of log-transformed *K_p_* and *K_d_* parameters with marginal density plots for each subject in session 1. Best-fitting linear regression line is shown. Log transformation corrected the right skew.
